# Loot

**Published:** 1869-03-15

**Authors:** 


### LOOT.

### *A Practical Point in the Treatment of Throat Diseases.*

BY FREDERICK A. BURRALL, M.D.

"It is often a difficult matter to examine satisfactorily the throats of patients who are lying in bed. The head of the bed may be towards the window, thus placing the patient's mouth away from the light, and the glare of a lamp held before the face is often painful to the eyes of the sick. Sitting up in bed and twisted towards the light is a constrained, and, to a debilitated invalid, an exhausting position; and while a child would be willing to open its mouth, it would often rebel against sitting up for a throat examination. The physician, conscious that his patient is in a fatiguing attitude, hurries his investigations, and sometimes obtains but a perplexing view. These annoyances may be lessened or obviated by the use of a concave mirror, with a focal distance of about twelve inches. Daylight can be reflected into the throat of the patient, while he lies quietly in bed or slightly raised on pillows, and the lamp used for illumination at night can be placed above or at the side of his head. Of course it is well-known that such mirrors are used by those who are constantly treating diseases of the throat, but the object of this article is to recommend them to more general use. Much weariness would thus be spared the sick, and such a mirror is also useful for the examination of any cavity on a day or at night."—*Medical Gazette*.

### *The Case of Dr. Earle.*

Our lively contemporary of the *Gazette* thus discourses:

"Tony Weller's impressive warning to his son: 'Samivel, beware of widders,' has received striking corroboration in Chicago, in the experience of Dr. Earle, of that city. His 'captiwation' in matrimony some years ago by a female of that description, was followed, according to the custom of the community in which they resided, by a divorce. Not having learned that to make overtures towards reconciliation to a woman who has injured one, is invariably considered by her as an unpardonable outrage, he imprudently sought to revive her affection; and roused to revenge by this crowning injury, she had him arrested on charges compared with which the wildest imaginings of 'blood and thunder' romancists sink into insignificance. Mutilated infant corpses in bureau drawers, or used as fuel in his office grate; smothered wails of infantile anguish issuing from unexpected corners; truculent boasts of his murderous exploits, and gleeful demonstrations of their details; these are some of the items of the tale she tells, and as its consequence Dr. Earle is now in prison. The whole question hinges on the credibility of female testimony, and we think that as a general formula it may may be stated that a woman's veracity is in inverse ratio to her malevolence."

### *Prof. Halford's Treatment of Snake Bites.*

Prof. Halford, of Australia, has instituted a number of experiments on animals, with the view of discovering an agent which would avert the poisonous effects of the bites of venomous snakes. The subject, it is stated (*Lancet*, Jan. 30, 1869), has recently attracted a large amount of attention in that country, owing to the professor having employed his remedy—a solution of ammonia injected into the veins—with success in the case of a man exhibiting all the symptoms of snake poisoning in a dangerous degree. "A man was bitten by a venomous snake which he had taken into his hands, supposing it to be dead. Not long afterwards he became drowsy, and mentioned the occurrence to one of his mates. The latter immediately set about procuring medical assistance, but by the time it arrived the man was comatose, and his lower extremities paralyzed. Galvanism and other usual remedies were applied but without effect. In this extremity, the medical man first called in caused Dr. Halford to be telegraphed for. The case was the first opportunity he had had of applying his new treatment to a human being, and he at first felt some hesitation in resorting to it. An incision, however, was made through the skin, exposing the superficial radial vein, and the point of the syringe being introduced into the vein, the injection (of ammonia) was completed. The beneficial effect was immediate. From an almost pulseless state, and from a stupor verging on death, the patient speedily beand he is now reported to be nearly well. Whether the earlier treatment in any way contributed to the cure cannot, perhaps, be certainly known, but there appears to be little came conscious. He has been steadily recovering since, doubt—the medical men entertain none—that the case must have ended fatally but for Professor Halford's treatment. We require, of course, some further cases before the merit of the discovery can be determined. Ammonia is not a new remedy for snake bites, but Dr. Halford has the credit, unquestionably, of having applied it in a direct way by injection into the blood, so that its effects should be immediate and general. Its application in this way could only be made safe in skilled hands. The discovery was not fortuitous, but resulted from a consideration of the microscopical alterations which he found taking place in the blood vessels of animals subjected to the snake poison. It is only right to add, however, that the observations of Dr. Fayrer and others have failed to verify the statements made by Dr. Halford on the altered conditions of the blood-corpuscles.* We learn from a letter which Dr. Halford addressed to the *Melbourne Argus*, that he had previously instituted a series of experiments on the lower animals, and he publishes five of them which he performed on dogs. In four instances the treatment was successful. To carry out the treatment a solution of ammonia—of the strength of one part of strongest liquor ammoniæ to two parts of distilled water—and an ordinary hypodermic syringe are required. The ammonia is thrown directly, but gradnally, into the blood by puncturing any superficial vein, and may be repeated as its beneficial operation ceases."

Three other cases treated by Prof. Halford's plan are quoted in the *Med. Times and Gazette*, Jan. 30, 1869, one of which we will quote, as showing more clearly Prof. Halford's treatment:

"On November 11, a man came under my care, says Dr. Dempster, of Beechworth, for treatment for the bite of a

---

* Dr. S. Weir Mitchell, in a note to us, writes: "Dr. Halford's treatment is a perfectly rational means of rousing an enfeebled heart. Ammonia given by the mouth did not seem to me as efficacious as alcohol. Perhaps the latter might be used subdermically, or thrown into a vein with success where speedy effects are desirable. Dr. Halford is himself inclined to abandon the view he formerly held as to the condition of the blood in snake bites. The cause of the hæmorrhage after snake bites and of the widely distributed local extravasations, has been explained by me in the *N. Y. Med. Journal.*"

blacksnake. He was bitten about 8 A. M., and after several minutes had elapsed he pricked and incised the wound. Before this, however, he felt very giddy. I did not see him for more than an hour afterwards, and treated him in the usual way, with brandy and ammonia, and scarifying the wound and applying ammonia. The man, however, fell into a state of stupor, and when I was called to him at midday, we could not rouse him. I therefore injected liq. ammon. fort. into the saphena vein, and also hypodermically. This affected him at once, and after the second injection he woke up, and became sensible; his pupils, which had before been very sluggish, acted well; and his pulse rose from fifty-six to seventy. After this he went on well, with the exception of violent vomiting for twelve hours. He is now (November 15) convalescent, but very weak. I, of course, continued the stimulant treatment, but I certainly attribute the man's recovery to the injection of ammonia, of which I altogether injected about twelve minims. Professor Halford mentions, with reference to this case, that the liq. ammon. fort. should be diluted before injection in such cases with two or three times its quantity of water, and of this mixture from twenty to thirty drops should be injected into one of the larger veins. The syringe, he adds, should be carefully introduced, so as to give the ammonia a fair chance. He disapproves of merely throwing the injection under the skin, and believes that after the injection of the ammonia there is no necessity for resorting to the use of stimulants. In a matter so important as this, the result of a few more experiments will be looked forward to with great interest by the public generally, as they will no doubt be by the profession."—*Med. News.*

### *Strength of Carbolic Acid Solutions.*

"In view of the fact that carbolic acid is now largely in use in medicine, with a probability that its range of application will be increased, it is well for prescribers to be very careful of the particular preparation they employ. Instances are reported where much damage has been done by the external application of this substance in solution, and we ourselves have seen carbolic acid ordered from the apothecaries, in such a way as to evince plainly the fact of a most blissful ignorance of whether the medicine was a solid or a fluid, or in what proportions it was proper to use it. Dr. W. T. Channing, of Providence, reports to the Boston *Journal of Chemistry* several cases of serious results, from the use of the concentrated fluid acid, which is dispensed by some under the name of 'solution carbolic acid,' when the prescribers intended only a milder solution, which they had been in the habit of using, but had obtained it from other druggists. Until, therefore, some distinctive nomenclature shall be given to the various preparations of this substance, and some official 'solution' shall be decided upon, physicians cannot be too careful in learning the strength of the solution employed, and it would be advisable to give explicit directions where to procure it."—*Philadelphia Reporter.*

### *Sea-Sickness.*

The following rules are laid down by Prof. Fordyce Barber against Sea-sickness:

1. Have every preparation made at least twenty-four hours before starting, so that the system may not be exhausted by overwork and want of sleep. This direction is particularly important to ladies. 2. Eat as hearty a meal as possible before going on board. 3. Go on board sufficiently early to arrange such things as may be wanted for the first day or two, so that they may be easy of access; then undress and go to bed, before the vessel gets under way. The neglect of this rule by those who are liable to sea-sickness is sure to be regretted. 4. Eat regularly and heartily, but without raising the head for one or two days. In this way the habit of digestion is kept up, the strength is preserved, while the system becomes accustomed to the constant change of equilibrium. 5. On the first night out take some laxative pills, as, for example, two or three of the compound rhubarb pills. Most persons have a tendency to become constipated at sea, although diarrhæ occurs in a certain per centage. Constipation not only results from sea-sickness, but in turn aggravates it. The reason has already been given why cathartics should not be taken before starting. The effervescing lazatives, like the siedlitz, or the solution of the citrate of magnesia, taken in the morning on an empty stomach, are bad in sea-sickness. 6. After having become so far habituated to the sea as to be able to take your meals at the table and go on deck, never think of rising in the morning until you have eaten something, as a plate of oatmeal porridge or a cup of coffee or tea, with some sea-biscuit or toast. 7. If subsequently, during the voyage, the sea should become unusually rough, go to bed before getting sick.—*Philadelphia Reporter.*

### *Wombless Women.*

(The subjoined is an extract from an article entitled *Surgical Cases, Devon and Exeter Hospital*, found in the last volume of *St. Bartholemew's Reports:*)

That an incapacity for menstruation should consist with female health, is a strange fact. In two of the three following cases of wombless women, the defect was congenital.

CASE 1.—About nine years since, I removed the womb by ligature. In the "Address in Surgery" of 1860, I gave this case very circumstantially. The main facts were, that the patient was twenty-one; that it was her first labor; that she was delivered while standing; that the midwife tugged at the naval string; that the womb was everted; and that she nearly bled to death on the spot. After many months, when she was nearly exhausted by recurring hemorrhage, I tied the womb with Gooch's canulæ.

As I was going into the operating room, the nurse, a worthy old woman and a favorite, said, "Please, sir, if you take out her womb, I suppose she can never have her courses again, and if so, what then?" I replied, "Nurse, if I do not take out her womb, she may die before night. If I can I will save her life now. That is my present thought. Icannot look beyond that."

After all manner of perils, she made a thorough recovery. Of course she has never menstruated since, but when I saw her, three years after, she was in good health and good spirits. I know that she was perfectly well several years after that. Her whole aspect is feminine, her complexion is fair and delicate, as it has always been, but in no wise chlorotic. She walks with her young man like any other servant girl; she cares nothing for the loss of her womb, which seems to have produced no other effect, moral or physical, than the cessation of her menses. That is the answer to the nurse's question, 'if so, what then?'

CASE 2.—About four years ago, a very delicate young woman, aged twenty-five, evidently in declining health, came under my charge at the hospital. She told me that she had been left an orphan, (I believe one, at least, of her parents had died of consumption), that she was delicate, and had not been able to retain her water like stronger girls; but that she had been brought up carefully; was now the mistress of a national school, and had married at twenty-three. She then added that coition had, from the first, been accompanied by pain, which, instead of diminishing, as she expected, had increased, until she could not bear it; and that her urine escaped from her involuntarily. She and her husband, who were reputed to be very worthy young people, and a most attached couple, were greatly distressed, as they well might be. I learnt then, also, that she had never menstruated,

Her pudenda were perfectly natural. About three inches up the vagina there was a sudden narrowing, like a stricture. Its circular edge was soft, and the orifice sufficiently open to admit the finger; but it could have been easily dilated to a greater extent. It exerted a gentle compression.

This was the sphincter, and beyond it was the bladder. There was no trace of a womb.

I told her that sexual intercourse must cease forever. She was much grieved on her husband's account; of whose regard for her she spoke with genuine feeling. I recommended tonics, and she went home.

I heard nothing of her until lately, when she and her husband called on the nurse. They appeared a fond couple still. Her health was restored; and the incontinence of urine not worse than when she was a child.

Case 3.—I was consulted in the case of a lady's maid, aged forty, who previous to the last six weeks, had enjoyed excellent health. She was a neat little figure, a brunette, must have been pretty, and was young looking for her age. She had a swollen knee, and flying rheumatic pains, for which she could not account, but she was chiefly harrassed by an irritable bladder. Micturition was frequent, scarcely to be restrained, and painful, both at the time and afterwards. She had used a hip-bath and opiate fomentations, with partial relief. I was to ascertain if a urethral wart or a calculus occasioned her distress.

Her urine had always been apt to escape when she coughed, but she had not been subject to leucorrhœa, and she never menstruated.

The external appearance of the genitals had nothing peculiar. The pubes and the labia were natural; the glands of the clitoris and the nymphæ were fully developed; but I could not find the urethra.

The vagina was such as might be expected in a virgin, except that there was neither transverse rugæ, nor hymen, nor caranculæ, nor fossa navicularis. It opened abruptly, just in front of the perineum. It admitted the finger, which, when it had penetrated somewhat less than three inches, came upon a dimple. This yielded, circularly, on gentle pressure, and the finger entered the bladder. As it was withdrawn, the sphincter closed again. The bladder was not set on obliquely like a womb; but quite at the end of a canal, which was no vagina at all. It was simply a preternatural urethra applied, as in the male, against the rectum. There was not the slightest trace of an os uteri or cervix—neither could any womb or corresponding body be felt from the bowel.

She told me that, some years before, she had been engaged to a young man, who died. Spoke of him, as a woman should, tenderly and regretfully; but did not affect to represent her love for him as Platonic.

Judging from these cases, the menstrual discharge is not necessary to the health of a woman who has no womb. In the intervals between one pregnancy, or lactation, and another, it would appear that the presence of a womb has a special and accumulating influence on the constitution; and that it is thus the organ which, by its own periodical discharge, affords that relief to the whole frame, of which it has itself created the necessity.

It is the womb alone, and not the whole generative apparatus, or, indeed, any part of it. Looking at their personal appearance, their views, manners and habits, and their womanly, albeit their correct and estimable feeling toward the men they loved, I believe the subjects of the cases 2 and 3 to be real women—saving their wombs. If it be contended that their ovaries may likewise be wanting, I can only reply that in case 1 the generative system was undoubtedly complete, for that woman lost her womb, and nothing else, after bearing a child; and yet she is as healthy, without menstruation, as the two others, who never had a womb."—*Western Med. Jour.*

### *India-rubber Sponge.*

This seems a very ingeniously contrived substance. It is an artificial sponge, made apparently by filling India-rubber in a fluid state with bubbles of gas, and then allowing it to harden, the result being a mass of India-rubber as full of holes and pores as a French roll. It is very elastic, will take its fill of water easily, and may be used for many purposes, instead of stiff sponge—as, for instance, in the bath as a flesh-brush, for cleaning paint, windows, etc. But it also seems capable of being made into pads for fracture, hernia, etc., and if so, its lightness, pliability, and elasticity may make it of great service.—*Med. Times and Gazette.*

